# Outcomes of tissue reconstruction in distal lower leg fractures: a retrospective cohort study

**DOI:** 10.1186/s12891-020-03827-9

**Published:** 2020-12-01

**Authors:** Emrah Aydogan, Stefan Langer, Christoph Josten, Johannes Karl Maria Fakler, Ralf Henkelmann

**Affiliations:** Department of Orthopedics, Trauma and Plastic Surgery, University of Leipzig, Liebigstraße 20, 04103 Leipzig, Germany

**Keywords:** EQ-5D-5L, Flap coverage, Foot and ankle outcome score, Infection, Lower leg fracture

## Abstract

**Background:**

Open and closed fractures can be associated with posttraumatic or postoperative soft tissue defects caused by initial trauma, operative procedures, or infections. This study evaluated the postoperative outcomes in patients with open or closed lower leg fractures, related soft tissue defects, and subsequent flap coverage.

**Methods:**

We performed a retrospective single-center cohort study in a level 1 trauma center. We analyzed the patients treated from January 2012 through December 2017 and recorded demographics, treatment, and outcome data. The outcome data were measured via patient-reported Foot and Ankle Outcomes Scores (FAOS) and EQ-5D-5L scores.

**Results:**

We included 22 patients with complicated fractures (11 open and 11 closed) and subsequent soft tissue defects and flap coverages. The mean follow-up time was 41.2 months. Twenty-one patients developed infections, and necrosis at the site of surgery manifested in all closed fractures. Therefore, all patients needed soft tissue reconstructions. Preoperatively, 16 patients underwent arterial examinations via angiography and six underwent ultrasound examinations of the venous system. Ten patients had complications involving the flaps due to ischemia and consequent necrosis. The mean EQ-5D index was 0.62 ± 0.27, and EQ-5D VAS score was 57.7 ± 20.2. The mean FAOS was 60.7 ± 22.2; in particular, quality of life was 32.3 ± 28.8. The rate of returning to work in our patient group was 37.5% after 1 year.

**Conclusions:**

Distal tibial fractures often require revisions and soft tissue reconstruction. The evaluated patient population had poor outcomes in terms of function, quality of life, and return to work. Furthermore, patients suffering from flap ischemia have worse outcomes than those without flap ischemia.

## Background

Complex distal lower extremity fractures are frequently associated with soft tissue injuries that require challenging and substantial reconstructions [1]. Therefore, it is of high importance for physicians to identify the right course of treatment. Among available options, bone and soft tissue reconstruction and limb rescue both require long-term treatment and have more potential comorbidities than primary amputation [2]. Complicated fractures can be limb-threatening if the arterial flow is reduced because of swelling, trauma, or compression. Arterial injuries have resulted in significant complications for patients with lower extremity fractures requiring flap coverage; however, limb salvage is still effective in most cases [3].

Before performing definitive reconstruction, surgeons must examine and determine the extent of injury and the perfusion status of the affected limb. In open fractures, prompt coverage after bone stabilization reduces the complication rate of open fractures [4–6]. In a case series regarding classification schema, Gustilo et al. described patients with ischemic limbs requiring emergency revascularization (type IIIC) as having the worst prognosis of all patients requiring limb salvage [6]. Traumatic ankle injuries are known to impact patient-reported function and quality of life [7, 8]. However, little is known about patient-reported outcomes in people with severe injuries that require revisions and soft tissue reconstruction.

This study investigated the outcomes of qualities of reconstruction with lower leg fractures of patients who required free-flap coverage in order to determine the relationship between the postoperative outcomes and qualities such as mobility, pain, recovery, sports, and the quality of life.

## Methods

### Study aim, setting, and design

We performed a retrospective monocentric study at a level 1 trauma center with the aim of evaluating postoperative outcomes in patients who required soft tissue reconstruction for lower leg fractures. Patient-reported outcomes, including quality of life (QoL) and the ability to return to work, were assessed via questionnaires. Follow-up scores were evaluated in the outpatient clinic, via telephone interview, or via declarations and forms mailed to and completed by patients. A minimum follow-up of 12 months was set as standard. This allows a more differentiated analysis of the outcome.

### Participants and materials

We identified 22 patients who were treated surgically for lower leg fractures and underwent soft tissue reconstruction via plastic surgery from January 2012 through December 2017 by querying the hospital database and using the International Classification of Disease (ICD) code for both fractures and flap procedures (e.g. S82.5 + 5–905.0f). To avoid including patients with improper codes and those who did not fulfill our inclusion criteria, all patient charts were screened manually. All variables that were to be recorded were specified in advance in a pre-prepared spreadsheet. Inclusion criteria were patients older than 18 years of age who had sustained a traumatic open/closed lower leg fracture, localized as code 43 or 44 according to the AO/OTA classification (*Arbeitsgemeinschaft für Osteosynthesefragen* Foundation/Orthopaedic Trauma Association classification), primarily treated with bone stabilization and secondarily with one or more flaps for tissue reconstruction. Every patient has the same opportunity to receive physical therapies as recommended by the surgeon. Every patient was given the same recommendation and prescription for physical therapies of equal intensity and frequency.

### Descriptive and outcome measures

Standard parameters that we collected were age, sex, and body mass index (BMI). Comorbidities were also recorded and categorized into four groups according to the number of comorbidities as follows: no comorbidity, 1–3 comorbidities, 4–5 comorbidities, and ≥ 6 comorbidities. Comorbidities were defined as diabetes mellitus, arterial hypertension, coronary heart disease, heart failure, asthma, COPD, emphysema, any type of tumor disease, a second tumor as an independent comorbidity, apoplexy with residuals in the history, neurological pre-existing conditions such as multiple sclerosis, rheumatic diseases, organ transplantation, congenital immune defects, HIV, cirrhosis of the liver, and kidney failure requiring dialysis. Diabetes mellitus, nicotine abuse, and alcohol/drug abuse were listed separately as nominal scale variables. Accompanying injuries to the affected ankle joints were classified as closed or open fractures (open fractures with soft tissue damage were assigned a score > 1, as indicated by Gustilo and Anderson) [5]. Fracture morphology was classified according to the AO/OTA criteria [9]. Complications of osteosynthesis and flap coverage were reported. The results of preoperative vascular diagnostic tests, consisting of Duplex ultrasound of the venous system and interventional angiography of the arteries, were categorized as binary variables.

Postoperative outcomes were measured using two different patient-reported outcome scores: The FAOS and its corresponding subscores is a 42-item questionnaire – including symptoms, pain, function in daily living / activities of daily living (ADL), function in sports and recreational activities (sport/rec), and QoL. The EQ-5D-5L score is a well-validated generic health-related QoL instrument [10–13]. The EQ-5D-5L is a Patient Reported Outcome (PRO) Score that uses 6 questions to assess the quality of life of patients in general, regardless of their disease. It also includes a visual analogue scale (VAS, 0–100 points) and a descriptive EQ-5D-5L system that considers the following dimensions (mobility, self-care, general activities, pain / physical complaints, fear / dejection).

Regarding the FAOS score (maximum, 100), a lower score represented more symptoms or pain, greater difficulty performing ADL and sport/rec, and poorer QoL. This rating is also valid for the EQ-5D VAS (maximum, 100).

### Statistical analysis

Findings are reported by the mean value for continuous data (standard deviation between parentheses) and number for categorical data (percentage between parentheses). T-tests for continuous variables was performed between the subgroup flap ischemia yes vs. no.. All tests were two-sided, and statistical significance was set at *p* < 0.05. Analyses were performed using IBM SPSS Statistics version 24 software.

## Results

### Participants

We identified 22 patients according to our inclusion criteria with a follow-up of 41.3 month (range 22–84 month). Eighteen patients returned the questionnaires. Of the four non-responders, one patient had died, one was not able to respond because of a mental disorder, and two were not interested in responding. All data concerning descriptive information on the patient sample are shown in Table 1. Moreover, results concerning occupational outcomes of surgery are the following. Before their trauma, 11.1% of patients were pensioners, 22.2% were unemployed, and 67.5% were employed. Of those who were employed, 37.5% returned to work. After returning to work, 50% of the patients had persistent symptoms and 50% reported limitations at work due to their injuries.

**Table 1:** Descriptive data of the study cohort

**Continuous variables**			
	**Mean**	**SD**	**Range**
**Age (years)**	58.0	15.9	17–81
**BMI (kg/m**^***2***^**)**	27.6	6.5	19.1–42.5
**Categorical variables**			
		**N**^**1**^	**%**
**Sex**	m	14	63.6
	f	8	36.4
**Smoking**	Y	12	54.5
	N	10	45.5
**Substance Abuse**	Y	8	36.4
	N	14	63.6
**Diabetes Mellitus**	Y	5	22.7
	N	17	77.3
**Comorbidities**	None	7	31.8
	1–3	10	45.5
	4–5	4	18.2
	> 6	1	4.5

Abbreviations: *BMI* body mass index, *f* female, *m* male, *N*^*1*^ number, *N* no, *SD* standard deviation, *Y* yes

### Fracture types and complications.

Fractures were classified according to the AO/OTA classification and to the criteria by Gustilo and Anderson [5]. Fracture classification and primarily used flaps are shown in Table 2.

**Table 2:** Classification of fractures

		N^**1**^	%
**Classification AO/OTA**	**44 A1**	3	13.6
	**44 A2**	2	9.1
	**44 A3**	1	4.5
	**44 B1**	3	13.6
	**44 B3**	5	22.7
	**44 C2**	1	4.5
	**43 A3**	3	13.6
	**43 C3**	3	13.6
	**43 C1**	1	4.5
**Open fracture**	**Y**	11	50
	**N**	11	50
**G&A Classification**	**1**	0	0
	**2**	3	13.6
	**3a**	2	9.1
	**3b**	6	27.3
**Flaps**	**ALT**	15	68.3
	**Latissimus dorsi**	5	22.7
	**TDAP**	1	4.5
	**Soleus**	1	4.5

Abbreviations: *AO/OTA* Arbeitsgemeinschaft für Osteosynthesefragen, *ALT* anterolateral thigh flap, *G&A* Gustilo and Anderson, *N1* number, *TDAP* thoracodorsal artery perforator, *N* no, *Y* yes

All closed fractures developed necrosis and surgical site infection (SSI) and required subsequent revisions and debridement, resulting in larger soft tissue defects. In 15 of 17 instances of SSI, bacterial contamination was detected (Table 3).

**Table 3:** Complications and infections

Complication		N^**1**^	%
**Osteosynthesis**	Y	17	77.3
	N	5	22.7
**Flap ischemia**	Y	13	59.1
	N	9	40.9
**SSI**	Y	22	100
	N	0	0
**Early SSI**	Y	12	54.5
	N	10	45.5
**Late SSI**	Y	10	45.5
	N	12	54.5
**Bacterium**	*Staph. aureus*	4	18.2
	*Staph. epidermidis*	3	13.6
	*Staph. capitis*	1	4.5
	*Strep. pyogenes*	1	4.5
	*E*^*1*^*. coli*	1	4.5
	*E*^*2*^*. faecalis*	1	4.5
	*E*^*3*^*. cloacae*	3	13.6
	*Bacillus cereus*	1	4.5
	no bacterial detection	7	31.8

Abbreviation: *E1* Escherichia, *E2* Enterococcus, *E3* Enterobacter, *N1* number, *N* no, *SSI* surgical site infection, *Staph* Staphylococcus, *Strep* Streptococcus, *Y* yes

Preoperatively, interventional arteriography was performed in 16 patients and Duplex ultrasound of the venous system was performed in six of those 16 patients without findings of thrombosis. In seven patients, adequate circulation in all three arteries of the lower leg was found. In five patients, arterial stenosis was treated by balloon dilation. A venous bypass graft was applied in one patient. In three patients, treatment with angiography and recanalization via stenting of the occlusion was performed after loss of the first flap. Complications occurred in 12 flaps; in 10 of those cases, the flap was lost due to perfusion problems and necrosis. As a result, revision flap coverage was required. The mean number of revision surgeries was 7.63 ± 4.98 (range 2–22). None of our patients reported problems at the donor sites after soft tissue grafts such as wound healing disorders, pain, or mobility limitations.

### Patient-reported outcomes

The results of FAOS and EQ-5D are given in Table 4. Most patient complaints were revealed in the “quality of life” subscore (mean score 32.3) (*p* > 0.05). The “symptoms” subscore mean was moderate at 60.1 (*p* > 0.05), and patients were bothered the least by pain with a mean score of 73.6 (p > 0.05).

**Table 4:** Outcomes: FAOS, FAOS subscores, and EQ-5D VAS scores

	Mean	SD	Minimum	Maximum
FAOS	60.7	22.2	27.00	100
Symptoms	60.1	27.5	21.43	100
Pain	73.6	23.0	30.56	100
ADL	66.9	19.7	38.24	100
Sport/Rec	40.4	39.1	0.0	100
QoL	32.3	28.8	0.0	100
EQ-5D VAS	57.7	20.2	30.00	100
EQ-5D index	0.6215	0.279	0.13	1

Abbreviations: *ADL* activities of daily living, *FAOS* Foot and Ankle Outcome Score, *QoL* quality of life, *SD* standard deviation, *Sport/Rec* function in sports and recreational activities, *VAS* visual analog scale

Two patients with amputations were included in the follow-up. One of those two patients underwent bilateral amputation after 22 revision surgeries; he reported an EQ-5D VAS of 50 and an EQ-5D index of 0.26. The other patient experienced bilateral trauma and underwent direct amputation on one side and seven surgeries on the other side; he reported an EQ-5D VAS of 80 and an EQ-5D index of 0.91.

Furthermore, we analyzed the outcome of the 10 patients with flap ischemia in comparison with the other patients. In our follow-up examinations we determined that patients with flap ischemia had poorer outcomes than patients without ischemic complications. Detailed FAOS subscores and EQ-5D VAS related to ischemic and non-ischemic groups are shown in Table 5. Patients with ischemia were found to be lower in all subscores compared to patients without ischemia. The lowest subscore was QoL (mean 21.88) (*p* > 0.05).

**Table 5:** Ischemia of flaps

	yes				no				*p*
	Mean	Minimum	Maximum	SD	Mean	Minimum	Maximum	SD	
Follow-up time in months	43.50	26.00	80.00	18.27	39.42	22.00	84.00	17.39	
Pain	69.45	30.56	94.44	26.47	77.78	47.22	100.00	20.64	0.6
Symptoms	46.43	25.00	71.43	19.56	73.81	21.43	100.00	28.72	0.08
ADL	61.52	38.24	94.12	19.50	72.31	38.24	100.00	20.04	0.4
Sport/Rec	29.17	5.00	85.00	34.56	51.67	0.00	105.00	43.09	0.3
QoL	21.88	0.00	56.25	20.44	42.71	6.25	100.00	33.87	0.2
FAOS	53.00	27.00	81.00	20.09	68.33	31.00	100.00	23.32	0.3
EQ-5D VAS	59.17	35.00	100.00	25.38	56.43	30.00	80.00	16.51	0.8

Abbreviations: *ADL* activities of daily living, *FAOS* Foot and Ankle Outcome Score, *QoL* quality of life, *SD* standard deviation, *Sport/Rec* function in sports and recreational activities, *VAS* visual analog scale

## Discussion

This study showed the course of treatment in a highly selective patient cohort with lower leg fractures treated with reconstruction and flap coverage. Furthermore, we aimed to detect postoperative complications and outcomes. For all 22 patients, complications such as SSI, necrosis, implant failure, the presence of an open fracture or other factors led to subsequent flap coverage. The bacterial spectrum described in this study was almost identical to that reported in national surveillance data [14].

The results of FAOS and EQ-5D index and VAS instruments revealed poor function and QoL in patients who experienced lower leg fractures requiring flap coverage. Furthermore, patients with flap ischemia showed the poorest outcomes in our cohort, as measured by FAOS, FAOS subscores, EQ-5D index, and VAS.

Van Bergen et al. stated that the minimal detectable changes for each FAOS subscore were 17.1–20.8 at the individual level and 2.0–2.4 at the group level in the validation of the German version of the FAOS [9]. In a recent review, the FAOS was shown to be a reliable tool for the re-evaluation of ankle injuries and the assessment of recovery [15]. Considering these data, it is possible to compare our FAOS scores with results from other studies. Duckworth et al. reported a mean FAOS score of 76 (mean follow-up 6 years) in complex tibial pilon fractures, compared with the score of 60.7 in our study (mean follow-up 41 months) [7]. Unfortunately, no subscores were reported in the tibial pilon fracture study. Kent et al. evaluated unstable syndesmotic injuries with different treatment options [8]. Among three subgroups, the group with the worst outcome had the following results in the subscores: pain (89), symptoms (75), ADL (97), sport/rec (75), and QoL (44) [15]. In a study of 1670 patients with different ankle pathologies, despite comparable data, Golightly et al. found a significantly better QoL (83 points) [16]. In comparison to these studies, our patients had a particularly poor outcome.

Two previous studies with different types of ankle fractures found that the EQ-5D VAS score and EQ-5D index were significantly higher than those we observed in our current study with a mean EQ-5D VAS of 57.6 (30–100) and a mean EQ-5D index of 0.621 (− 0.205–1.000) [17, 18]. In our study, patients who required many revision surgeries were hospitalized for prolonged periods because of limb salvage with subsequent limited mobility. This may explain the poor QoL subscores. In a randomized study by Andersen et al. involving 97 patients with ankle injuries and follow-up at 2 years, much better EQ-5D VAS scores were reported, with a mean of 90 (75–95); however, none of the cases had any large soft tissue defects which required flap coverage [17]. In our study, each patient needed at least one flap coverage procedure because of large soft tissue defects. This may explain why the scores of our cohort were lower than the scores of the cohort of Andersen et al. [17]. Our study showed that severe injuries and complications requiring multiple revisions and flap coverage procedures had a negative impact on factors such as the quality of life.

The Lower Extremity Assessment Project (LEAP) study examined patients up to 7 years postoperatively and indicated poor physical and psychosocial results after lower limb trauma regardless of the initial treatment options (amputation or reconstruction). Both patient groups were severely disabled compared to the overall population [1]. Return to work was possible for 37.5% of our patients at the mean follow-up period of 41.2 months (22 to 84); however, according to the LEAP study, the return to work rate was 58% at 7-year follow-up [19]. Therefore, it may be expected that the return to work rate would increase in our patient group after 7 years. A meta-analysis revealed that limb reconstruction was at least as effective as amputation in terms of physical criteria such as the ability to perform ADL and recovery time required before returning to work [20].

Despite having undergone similar surgeries for comparable complications and conditions, the scores of the two amputees in our study showed great discrepancies relative to the other participants. These results may reflect the subjective perception of postoperative daily routines and patient status. These two patients were not able to go back to their professional life. EQ-5D VAS scores of these two patients were reported as 50 vs 80.

By focusing on a specific cohort, our study highlighted the peculiarities that differentiate these patients from those that undergo surgeries with lower rates of complications. However, focusing on a restricted sample was also a source of limitation for our work, as more detailed subgroup analyses could not be carried out. A second limitation was that this was a retrospective single-center design. Furthermore, no function-related examination such as strength measurement or similar was performed.

## Conclusion

This study primarily aimed to evaluate postoperative outcomes in patients who required soft tissue reconstruction for lower leg fractures. Our results showed that worse QoL, ADL, and other factors were observed in patients undergoing several surgical revisions due to postoperative complications. Furthermore, patients suffering from flap ischemia were shown to have worse outcomes than other patients. In addition, we showed that lower leg fractures with subsequent flap coverage as a result of complex injury and/or postoperative complications may require several surgical revisions.

## Data Availability

The datasets used and/or analyzed during the current study are available from the corresponding author on reasonable request.

## Abbreviations

ADL: Activities of daily living
AO/OTA: Arbeitsgemeinschaft für Osteosynthesefragen Foundation/Orthopaedic Trauma Association classification
*E. coli*: *Escherichia coli*
*E. cloacae*: *Enterobacter cloacae*
*E. faecalis*: *Enterococcus faecalis*
FAOS: Foot and Ankle Outcome Score
ICD: International Classification of Diseases
LEAP: Lower Extremity Assessment Project
QoL: Quality of life
SD: Standard deviation
Sport/Rec: Sports and recreation
SSI: Surgical site infection
*Staph*: *Staphylococcus*
VAS: Visual analog scale
